# Comprehensive Analysis of *TaNCED* Gene Family in Wheat Vernalization Process

**DOI:** 10.3390/biology14091293

**Published:** 2025-09-19

**Authors:** Guoqing Cui, Hao Cheng

**Affiliations:** 1 Center for Agricultural Genetic Resources Research, Shanxi Agricultural University, Key Laboratory of Crop Gene Resources and Germplasm Enhancement on Loess Plateau, Ministry of Agriculture and Rural Affairs, Taiyuan 030031, China; 2 State Key Laboratory of Crop Gene Resources and Breeding, Institute of Crop Sciences, Chinese Academy of Agricultural Sciences, Beijing 100081, China; 3 State Key Laboratory for Crop Stress Resistance and High-Efficiency Production, College of Life Sciences, Northwest A&F University, Yangling 712100, China

**Keywords:** wheat, *TaNCED*, gene family, abscisic acid (ABA), vernalization

## Abstract

9-*cis*-epoxycarotenoid dioxygenases (NCEDs), serving as the rate-limiting enzymes in abscisic acid (ABA) biosynthesis, play a pivotal role in regulating plant growth and development, as well as responses to abiotic stresses. Despite their agronomic importance, the molecular dialog between ABA signaling and vernalization, a cold-induced switch from vegetative to reproductive growth in wheat, remains poorly characterized, particularly regarding the *TaNCED* gene family members. Here, we systematically identified 13 *TaNCED* members in hexaploid wheat, followed by multi-omics characterization encompassing physicochemical properties, exon–intron architectures, conserved catalytic domains, protein motifs, and *cis*-acting elements. By analyzing transcriptome data from vernalization treatments, we profiled the expression patterns of *TaNCED* genes during vernalization. Notably, *TaNCED5-6A*, *TaNCED5-6B*, and *TaNCED5-6D* exhibited significant upregulation in vernalized leaves and tiller buds, while maintaining basal expression in the shoot apical meristem, the site of floral induction. This tissue-specific expression pattern implicates their specialized role in mediating vernalization responses via ABA biosynthesis. Collectively, our findings provide novel insights into the regulatory mechanisms of ABA-mediated vernalization in wheat and offer valuable targets for vernalization efficiency in cereal breeding programs.

## Simple Summary

This study investigates the role of 9-*cis*-epoxycarotenoid dioxygenases (NCEDs), key enzymes in abscisic acid (ABA) biosynthesis, during vernalization (cold-induced flowering) in wheat. A total of 13 *TaNCED* genes were identified in hexaploid wheat and then analyzed their properties, structures, and regulatory elements. Transcriptome data revealed that three *TaNCED5* genes (*TaNCED5-6A/6B/6D*) were significantly upregulated in leaves and tiller buds during cold treatment but remained minimally expressed in the shoot apical meristem (where flowering is initiated). This tissue-specific expression suggests these genes may mediate ABA-dependent vernalization responses. The findings advance understanding of ABA’s role in cold-induced flowering and provide potential targets for improving vernalization efficiency in wheat breeding.

## 1. Introduction

Abscisic Acid (ABA), a crucial phytohormone signal molecule, plays a central regulatory role in coordinating developmental processes and stress adaptation responses in plants [1]. Throughout the plant life cycle, ABA ensures adaptation to dynamic environmental conditions by precisely modulating key events, such as seed dormancy and germination [2,3,4,5], bud dormancy [6,7,8], stomatal movement [9], root development [10], and flowering time [11]. Particularly in abiotic stress responses, ABA acts as a signaling hub to rapidly activate downstream protective mechanisms, significantly enhancing plant tolerance to adversities such as drought, salinity, and low temperatures [12,13,14]. Therefore, elucidating the biosynthetic network and signal transduction mechanisms of ABA holds profound theoretical significance for uncovering regulatory principles of plant growth, development, and environmental adaptation strategies.

ABA biosynthesis is a multi-step enzymatic process orchestrated through coordinated interactions between plastid and cytoplasmic compartments, primarily occurring via the carotenoid pathway in plants to regulate cellular ABA levels. The carotenoid pathway initiates with pyruvate and glyceraldehyde-3-phosphate as substrates to generate the C_5_ precursor isopentenyl diphosphate (IPP) via the 1-deoxy-Dxylulose-5-phosphate (DXP) pathway. IPP condenses into the C_15_ farnesyl pyrophosphate (FPP), which is further polymerized into C_20_ intermediate geranylgeranyl pyrophosphate (GGPP). GGPP is then catalyzed by phytoene synthase (PSY) to produce C_40_ phytoene, which is subsequently converted into ζ-carotene, lycopene, β-carotene and then to a zeaxanthin through multiple dehydrogenation and cyclization reactions. During oxidative cleavage, zeaxanthin is catalyzed by zeaxanthin epoxidase (ZEP) to produce all-*trans*-violaxanthin, which is subsequently converted into 9-*cis*-violaxanthin or 9′-*cis*-neoxanthin and then specifically recognized and oxidatively cleaved by NCEDs to yield the C_15_ intermediate xanthoxin (XAN). Finally, xanthoxin is transported from the plastid to the cytoplasm, where it undergoes two-step catalysis oxidative reactions to produce ABA [15,16,17]. Notably, the NCED-catalyzed oxidative cleavage step serves as the rate-limiting bottleneck in ABA biosynthesis and is regulated by the *NCED* gene family members. Studies showed that *NCED* members often exhibit tissue-specific and spatiotemporal expression patterns, with members strongly induced by environmental stresses such as drought, cold, and salinity, directly influencing ABA accumulation [18,19].

Wheat (*Triticum aestivum* L.) is one of the most critical food crops around the world, supplying approximately 20% of dietary calories and protein for humans, with its yield stability directly impacting global food security [20]. Widely cultivated in temperate regions, winter wheat varieties have evolved a unique vernalization requirement mechanism to adapt to seasonal climate changes and avoid premature flowering before winter [21]. Vernalization refers to the physiological process wherein plants must undergo a prolonged period of low temperatures (typically 0–10 °C) to transition from vegetative growth to reproductive growth (flowering and seed set) [22]. Insufficient cold exposure results in prolonged vegetative growth and delayed heading date in winter wheat. Current research on the molecular mechanisms of wheat vernalization has identified a core regulatory network involving three key genes: *VRN1*, *VRN2*, and *VRN3* (*FT*) [23,24]. In the non-vernalized state, VRN2 protein represses the expression of the flowering promoter *VRN3*. Cold treatment induces the accumulation of *VRN1* (encoding a MADS-box transcription factor) in shoot apical meristems, which subsequently suppresses *VRN2* expression, thereby releasing VRN2-mediated repression of *VRN3* and ultimately promoting flowering [25,26,27]. Notably, vernalization not only regulates flowering transitions but also triggers complex physiological and biochemical changes. For instance, non-vernalized winter wheat exhibits sustained vegetative growth with increased leaves and tiller numbers. Concurrently, plants enhance cold tolerance under low temperatures by strengthening cell membrane stability, accumulating osmoprotectants, and inducing the expression of cold-responsive genes (e.g., *COR* genes), with ABA being a pivotal mediator of low-temperature responses and freezing tolerance in plants.

Although extensive studies have investigated *NCED* gene families and ABA’s role in cold stress responses in model plants, systematic characterization and functional analysis of the *TaNCED* gene family in wheat remain limited. Specifically, the regulatory network linking ABA biosynthesis mediated by *TaNCED* genes to wheat vernalization remains poorly understood. This study comprehensively identified 13 *TaNCED* gene family members in wheat, and analyzed their phylogenetic relationships, gene structures, conserved domains, motifs, and *cis*-acting elements. By integrating transcriptomic data from low-temperature vernalization with all *TaNCED* members, we characterized the expression patterns and potential functions of key *TaNCED* genes during vernalization. These findings provide novel insights into the integration of ABA biosynthesis with wheat vernalization responses and flowering regulation networks, offering valuable molecular targets for breeding stress-resistant and high-yielding wheat varieties.

## 2. Materials and Methods

### 2.1. Identification and Physicochemical Properties Analysis of TaNCED Members

The reference genome assembly data, protein sequences and gff3 annotation files of cultivar Chinese Spring (*Triticum aestivum* L.), maize (*Zea mays* L.) and foxtail millet (*Setaria italica* L.) were retrieved from the Ensembl Plants database (https://plants.ensembl.org/index.html, accessed on 1 May 2025). Reference protein sequences for NCED family members in *Arabidopsis thaliana* (AtNCED2, AtNCED3, AtNCED5, AtNCED6, AtNCED9) and *Oryza sativa* Japonica (OsNCED1-OsNCED5) (Supplementary Table S1) were obtained from the TAIR (https://www.arabidopsis.org/, accessed on 1 May 2025) and Rice Genome Annotation Project (RGAP) databases (https://rice.uga.edu/, accessed on 1 May 2025), respectively.

To systematically identify the *TaNCED*, *ZmNCED*, and *SiNCED* members, the protein sequences of AtNCEDs and OsNCEDs served as query templates against the protein database of wheat, maize and foxtail millet for homology-based genome-wide screening by BLASTP tool in TBtools-II (version 2.313) software [28]. The Hidden Markov Model (HMM) profile for the conserved domain (PF03055) of the NCED members was downloaded from the Pfam database (http://pfam.xfam.org/, accessed on 5 May 2025), and the HMMER tool [29] was then employed to search and compare the whole-genome protein sequences against these HMM profiles. The *TaNCED*, *ZmNCED*, and *SiNCED* members were also validated using the NCBI Conserved Domain Database (NCBI-CDD) tool to confirm the presence of characteristic NCED domains (https://www.ncbi.nlm.nih.gov/Structure/bwrpsb/bwrpsb.cgi, accessed on 8 May 2025) [30,31,32]. Combined the BLASTP, HMMER, and NCBI-CDD results, the members of the *TaNCED* gene family in wheat were further verified and annotated using the Triticeae-GeneTribe (http://wheat.cau.edu.cn/TGT/, accessed on 8 May 2025) with functional annotation [33].

The protein sequences of the TaNCED members were submitted to the “Protein Pa-rameter Calculator” module in TBtools to calculate key physicochemical parameters, including number of amino acids (AA), molecular weight (MW), theoretical isoelectric point (pI), instability index, aliphatic index and grand average of hydropathicity (GRAVY). The subcellular localization of TaNCED members were predicted by the Plant-mPLoc (http://www.csbio.sjtu.edu.cn/bioinf/plant-multi/, accessed on 10 May 2025) [34], DeepLoc 2.1 (https://services.healthtech.dtu.dk/services/DeepLoc-2.1/, accessed on 5 September 2025) and CELLO (http://cello.life.nctu.edu.tw/, accessed on 5 September 2025).

### 2.2. Construction of Phylogenetic Trees

To verify the evolutionary relationship of TaNCED, AtNECD, OsNCED, ZmNCED and SiNCED members, full-length protein sequences of these members were aligned using ClustalW with default parameters in MEGA 12 (version 12.0.11) software [35]. The neighbor-joining (NJ) method was used to construct a phylogenetic tree with the bootstrap value was set as 1000, Poisson correction model, and pairwise deletion. The online iTOL (http://itol.embl.de/, accessed on 12 May 2025) tool [36] was employed to further modify and visualize the phylogenetic tree.

### 2.3. Motif Composition, Conserved Domains and Gene Structure Analysis of TaNCED Members

The conserved motifs of TaNCED members were identified using the Multiple Expectation maximization for Motif Elicitation (MEME) database (https://meme-suite.org/meme/tools/meme, accessed on 15 May 2025) with the maximum number of motifs set to 10 and other parameters were set at default values [37]. The conserved domains of TaNCED members were predicted using the Batch CD Search tool on NCBI. The structure of *TaNCED* members, including the positions and numbers exon/intron and untranslated regions (UTRs), were determined according to genome annotation (gff3) files using the Gene Structure View (Advance) module in TBtools software. TBtools was employed for generating visual representations of the phylogenetic tree, motifs composition, conserved domains, and gene structures.

### 2.4. Chromosomal Location, Gene Duplication and Synteny Analysis of TaNCED Members

The chromosome mapping of *TaNCED* members were constructed using TBtools according to the positional information of these members retrieved from the reference gff3 files. Gene duplication events of *TaNCED* members were analyzed using MCScanX tool in TBtools-II (version 2.313), and then visualized using TBtools Advanced Circos tool. To investigate the selection pressure of *TaNCED* members during evolution, the KaKs Calculator tool in TBtools-II (version 2.313) software was employed to calculate the non-synonymous substitution rate (Ka) and synonymous substitution rate (Ks) of duplicated genes in the *TaNCED* gene family [38]. In addition, collinearity analysis of *TaNCED* members was conducted across wheat and other species (*Arabidopsis thaliana*, rice, maize, foxtail millet, *Triticum urartu*, *Aegilops tauschii* and *Triticum turgidum*) using the MCScanX tool with the default parameters (match score = 50, match size = 5, gap penalty = −1, overlap window = 5, e value = 1 × 10^−5^, and max gaps = 25), and then used the Dual Systeny Plot tool in TBtools for plotting.

### 2.5. Cis-Acting Elements Analysis of TaNCED Members

To investigate the expression regulatory landscape of *TaNCED* members, the promoter regions, defined as 2000 bp upstream of the initiation codon (ATG), were extracted from the wheat reference genome sequences. Then, the *cis*-acting elements within these promoter regions were predicted using the PlantCARE database (https://bioinformatics.psb.ugent.be/webtools/plantcare/html/, accessed on 20 May 2025) [39]. *Cis*-acting elements associated with plant growth and development, phytohormone responsiveness, as well as biotic and abiotic stress were identified and analyzed. Distribution maps of these *cis*-acting elements were generated using TBtools.

### 2.6. Spatiotemporal Expression Profiling of TaNCED Members During Vernalization Response

To investigate tissue-specific and temporal regulation of *TaNCED* genes during vernalization response, we analyzed RNA-seq expression profiles using public wheat transcriptome datasets (https://genomebiology.biomedcentral.com/, accessed on 30 May 2025) from vernalization experiments [40,41]. Expression levels were quantified as normalized TPM (Transcripts Per Million) values following cross-sample normalization, enabling comparative analysis of transcript abundance across six tissue types (leaf, vernalized leaf, axillary bud, vernalized axillary bud, shoot apex, and vernalized shoot apex) and three vernalization time points (V0, V28, and V28N6 days). This standardized approach allowed robust identification of differentially expressed *TaNCED* paralogs while controlling for technical variability between sequencing runs.

To investigate the tissue-specific and temporal regulation of TaNCED genes during the vernalization response, we analyzed RNA-seq expression profiles using publicly available wheat transcriptome datasets derived from vernalization experiments.

### 2.7. Dual-Luciferase Reporter Assays

The promoter region of *TaNCED5-6A*, *TaNCED5-6B*, and *TaNCED5-6D* (~2000 bp) were amplified from wheat genome DNA, and inserted into *pGreenII0800* vector to generate the reporter construct *35S::proTaNCED5-6A-LUC-35S::REN*, *35S::proTaNCED5-6B-LUC-35S::REN*, and *35S::proTaNCED5-6D-LUC-35S::REN*, respectively. Concurrently, the full-length coding sequence of the *VRN1-5A* was amplified from KN199 cDNA and inserted into the *pAN580-eGFP* overexpression vector to generate the effector construct *35S::VRN1-5A*. Both constructs were co-transfected into *Nicotiana benthamiana* and wheat leaf protoplasts via PEG-mediated transformation. Post-transfection (16–18 h), the firefly luciferase (LUC) and Renilla luciferase (REN) activities were measured using the Dual-Luciferase^®^ Reporter Assay System (Promega, E1960, Madison, WI, USA) on a microplate reader. LUC activity was normalized to REN activity to control for transfection efficiency. Primer sequences are listed in Supplementary Table S2.

## 3. Results

### 3.1. Identification and Molecular Features of TaNCED Members

Through a comprehensive genome-wide analysis, a total of 13 *TaNCED*, 7 *ZmNCED* and 4 *SiNCED* members were identified, and then systematically named according to their chromosomal locations (Supplementary Table S1). Physicochemical characterization analysis revealed considerable structural diversity among TaNCED proteins. The amino acids (aa) counts in these members ranged from 567 aa (TaNCED1-2A) to 643 aa (TaNCED5-6B), corresponding to molecular weights (Dalton) varied from 60,602 Da (TaNCED1-2A) to 69,262 Da (TaNCED5-6B), and the predicted theoretical isoelectric points (pI) spanned 5.41 (TaNCED3-5A and TaNCED3-5D) to 6.64 (TaNCED2-2B), indicating potential differences in solubility and charge-dependent interactions under physiological conditions. Biophysical parameters, including protein instability index (36.22 to 46.55), aliphatic index (74.18 to 86.9), and grand average of hydropathicity (GRAVY) values (−0.307 to −0.039) further highlight the physicochemical diversity within the TaNCED members (Table 1), which may underlie functional specialization or adaptation to specific cellular environments. Subcellular localization exhibits a certain degree of conservation among TaNCED members (Table 1). TaNCED1-2A and TaNCED1-2D showed exclusive localization in the chloroplast. TaNCED2-2A, TaNCED2-2B, TaNCED2-2D, TaNCED3-5A, TaNCED3-5D, TaNCED4-5B, TaNCED4-5D, TaNCED5-6A, TaNCED5-6B, and TaNCED5-6D were found to have dual subcellular localized in both the chloroplast and cytoplasm. Notably, TaNCED3-5B was the only gene with triple subcellular localization, distributed in the chloroplast, cytoplasm, and mitochondrion. The localization of TaNCED members in the chloroplast is consistent with their role in the chloroplast-based synthesis of ABA precursors. Dual localization in both the chloroplast and cytoplasm implies a dynamic regulatory mechanism. This suggests that ABA biosynthesis may be modulated through subcellular shuttling under stress conditions. Overall, the TaNCED gene family achieves spatiotemporal control over ABA synthesis through sequence diversification, flexibility in subcellular localization, and physicochemical adjustments, thereby enhancing plant resilience to environmental stresses.

### 3.2. Phylogenetic Analysis of NCED Genes Across Different Plant Species

**Figure 1:**
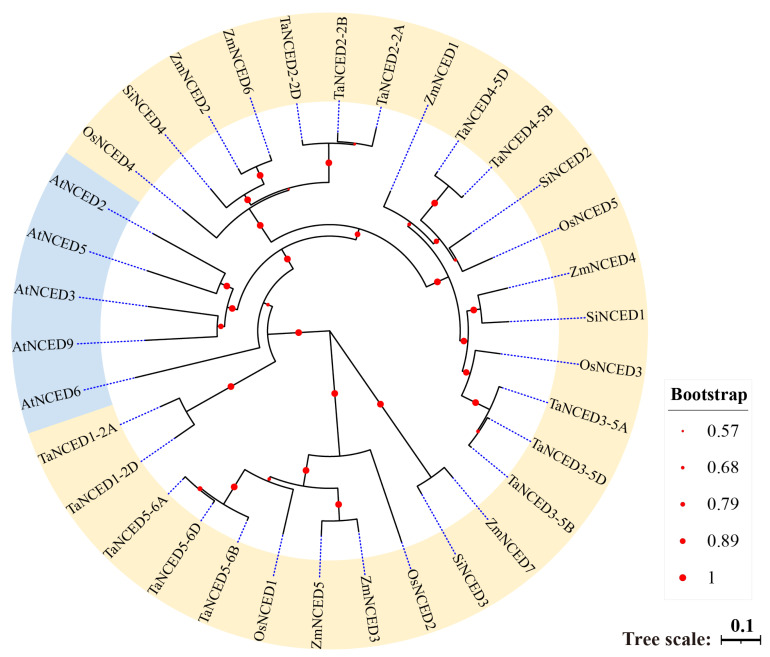
Phylogenetic analysis of NCED proteins were from *Arabidopsis thaliana* (At), *Triticum aestivum* (Ta), *Oryza sativa Japonica* (Os), *Zea mays* (Zm), and *Setaria italica* (Si). The tree was constructed using the neighbor-joining (NJ) method implemented in MEGA 12 (version 12.0.11).

The phylogenetic tree analysis reveals distinct evolutionary relationships among *NCED* genes from various species (such as *Arabidopsis thaliana* [At], *Oryza sativa* [Os], *Triticum aestivum* [Ta], *Zea mays* [Zm], and *Setaria italica* [Si]). The blue cluster primarily contains *NCED* genes from *Arabidopsis thaliana*, which forms a distinct branch separated from the *NCED* genes of other species, indicating that these *Arabidopsis NCED* genes have followed relatively independent evolutionary trajectories (Figure 1). In contrast, the yellow cluster encompasses *NCED* genes from multiple agronomically important crops, including rice, wheat, maize, and foxtail millet (Figure 1), suggesting that the *NCED* genes in these species had a close evolutionary relationship. This pattern implies that these *NCED* genes possibly shared a common ancestral origins followed by gene duplication and divergence events during evolution process, ultimately leading to the formation of orthologues genes in contemporary species. Overall, this phylogenetic tree clearly displayed the phylogenetic relationships of the *NCED* genes among these species, offering valuable insights for further investigating the functional conservation and divergence of these genes across different species.

### 3.3. Conserved Motifs, Domains, and Gene Structure Analysis of TaNCED Members

**Figure 2:**
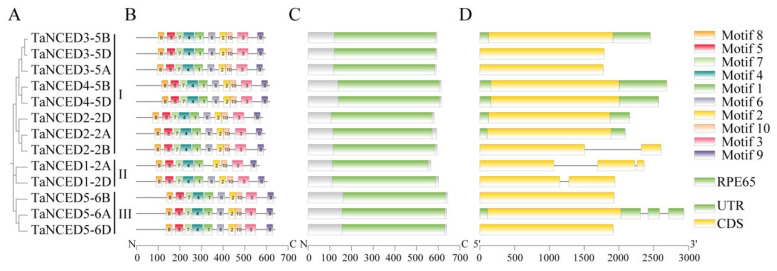
Comprehensive analysis of *TaNCED* gene family members. (**A**) Phylogenetic tree showing evolutionary relationships. (**B**) MEME-based visualization of conserved motifs, with colored boxes representing specific conserved amino acid sequences. (**C**) NCBI-CDD-predicted domain architecture, where colored boxes indicate functional conserved domains. (**D**) Gene structure analysis depicting exon–intron organization of *TaNCED* genes.

The examination of gene architecture provided deeper insights. To better understand the structural diversity of *TaNCED* members, the conserved motifs and domains were visualized according to genome annotation information. Firstly, an evolutionary tree was constructed for the *TaNCED* members using MEGA software, and categorized them into three types (Figure 2A). Conserved motif analysis revealed that the TaNCED gene family members share similar motif compositions, with 10 motifs (motif 1–10) distributed in a relatively conserved manner across all TaNCED members (Supplementary Figure S1), which indicates that these motifs may play important roles in the function of the TaNCED proteins. Notably, TaNCED1-2A uniquely lacking motif 6 (Figure 2B), and the absence of motif 6 in TaNCED1-2A suggests a potential isoform-specific functional modification, which is a finding warranting functional validation through site-directed mutagenesis. Furthermore, all TaNCED members harbored the typical conserved RPE65 domain (Figure 2C), a hallmark feature of NCED enzymes critical for 9-*cis*-epoxycarotenoid cleavage and unequivocally confirming their functional identity as NCED enzymes involved in carotenoid cleavage. Gene structure analysis revealed variation in exon number (1-3 exons), with most *TaNCED* members (*TaNCED3-5A/5B/5D*, *TaNCED4-5B/5D*, *TaNCED2-2A/2D* and *TaNCED5-6A/6B/6D*) possess a single exon architecture, while *TaNCED1-2D* and *TaNCED2-2B* contains two exons, and *TaNCED1-2A* uniquely contains three exons (Figure 2D). We also utilized the Wheat Expression Browser (https://www.wheat-expression.com/, accessed on 5 September 2025) and incorporated all available transcriptome datasets for analysis. We specifically focused on displaying high-level information, including tissue types, growth stages (age), and stress/disease treatment conditions. The results revealed substantial variations in the expression patterns among different TaNCED members (Supplementary Table S3). Although there is a high degree of conservation in motif composition, protein domains, and gene structure among the *TaNCED* gene family members, there are also some variations. For example, the lengths of the CDS regions and the positions of some motifs may differ among different members, which may be related to the functional diversification of the *TaNCED* gene family. Overall, the structural analysis of the *TaNCED* gene family members shows a high degree of conservation, which provides important insights into the functional conservation and evolution of this gene family.

### 3.4. Chromosomal Distribution and Collinearity Analysis of TaNCED Members

**Figure 3:**
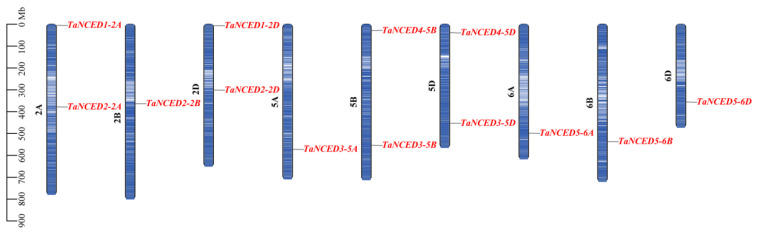
Chromosome distribution of *TaNCED* members in wheat.

The chromosomal distribution of *TaNCED* gene family members exhibit a distinct non-random pattern, with all 13 paralogs exclusively localized to chromosomes 2, 5, and 6 (Figure 3). Specifically, *TaNCED1-2A* and *TaNCED1-2D* are located on chromosome 2A and 2D, respectively. *TaNCED2-2A*, *TaNCED2-2B*, and *TaNCED2-2D* are located on chromosome 2A, 2B, and 2D, respectively. *TaNCED3-5A*, *TaNCED3-5B*, and *TaNCED3-5D* are located on chromosome 5A, 5B, and 5D, respectively. *TaNCED4-5B* and *TaNCED4-5D* are located on chromosome 5B and 5D, respectively. *TaNCED5-6A*, *TaNCED5-6B*, and *TaNCED5-6D* are located on chromosome 6A, 6B, and 6D, respectively.

The collinear relationships among the *TaNCED* gene family members show certain conservation, with some members having collinear relationships on homologous chromosomes (Supplementary Figure S2). For example, *TaNCED1-2A* on chromosome 2A has collinear relationships with *TaNCED1-2D* on chromosome 2D, while *TaNCED2-2A* on chromosome 2A exhibits collinear relationships with both *TaNCED2-2B* (chromosome 2B) and *TaNCED2-2D* (chromosome 2D). *TaNCED3-5A* on chromosome 5A has collinear relationships with *TaNCED3-5B* on chromosome 5B and *TaNCED3-5D* on chromosome 5D. *TaNCED4-5B* on chromosome 5B has collinear relationships with *TaNCED4-5D* on chromosome 5D. *TaNCED5-6A* on chromosome 6A has collinear relationships with *TaNCED5-6B* on chromosome 6B and *TaNCED5-6D* on chromosome 6D. These collinear patterns suggest that the *TaNCED* gene family may have expanded through polyploidization events during wheat evolution, with subsequent preservation of paralogs across homoeologous chromosomes. However, variations in collinear depth between subgroups (e.g., the chromosome 2A/B/D triad vs. chromosome 5A/B/D triad) indicate differential evolutionary constraints. The complete collinear observed in *TaNCED3-5A/B/D* and *TaNCED5-6A/B/D* subgroups contrasts with the partial collinear in *TaNCED1-2A/D* and *TaNCED2-2A/B/D*, suggesting functional diversification may have occurred post-duplication. This chromosomal organization and syntenic conservation provide insights into the evolutionary trajectories of ABA biosynthesis genes during allopolyploid wheat speciation. The Ka and Ks calculation results showed that the Ka/Ks of collinear gene pairs are both less than 1, indicating that they have been subjected to purification selection during evolution (Supplementary Table S4).

The synteny analysis between wheat (Ta) and *Arabidopsis thaliana* (At), rice (Os), maize (Zm), and foxtail millet (Si) was conducted (Supplementary Figure S3). There are certain synteny relationships between the *TaNCED* gene family members and the *NCED* gene family members of other species. For example, some *TaNCED* genes on wheat chromosomes 2A, 2D, 6A, and 6B have collinear relationships with *AtNCED* genes on *Arabidopsis thaliana* chromosomes 1 and 4. Similarly, there are extensive synteny relationships between *TaNCED* genes and *OsNCED*, *ZmNCED*, and *SiNCED* genes. These synteny relationships indicate that the *NCED* gene family has a certain degree of conservation during the evolution of different species and may have similar functions. In addition, the synteny relationships between different species also show some differences. For example, the synteny relationships between wheat and rice are more extensive than those between wheat and *Arabidopsis thaliana*, which may be related to the closer evolutionary relationship between wheat and rice. In conclusion, the interspecies collinearity analysis of wheat *TaNCED* gene family members reveals the evolutionary conservation and divergence of this gene family, which is of great significance for further studying the functions of *NCED* genes in different species.

Moreover, the analysis was conducted between wheat (Ta) and *Triticum urartu* (Tu), *Aegilops tauschii* (DD), and *Triticum turgidum* (AABB) (Supplementary Figure S4 and Table S5). There exist certain collinear relationships between the *TaNCED* gene family members and the *NCED* gene family members of these species. For instance, some *TaNCED* genes on wheat chromosomes 2A, 2B, 2D, 5A, 5B, 5D, 6A, 6B, and 6D exhibit collinear relationships with *TuNCED* genes on *Triticum urartu* chromosomes 2.0, 5.0, and 6.0. Similarly, there are collinear relationships between *TaNCED* genes and *NCED* genes in *Aegilops tauschii* (DD) and *Triticum turgidum* (AABB). These collinear relationships imply that the *NCED* gene family has maintained a certain degree of conservation during the evolutionary process across different species, and they might possess similar functions. Furthermore, the collinear relationships also display some variations among different species. For example, the collinear relationships between wheat and *Triticum turgidum* (AABB) are more extensive compared to those between wheat and *Triticum urartu*. This might be associated with the closer evolutionary relationship between wheat and *Triticum turgidum*. In summary, the interspecific collinearity analysis of *TaNCED* gene family members in wheat uncovers the evolutionary conservation and divergence of this gene family, which holds great significance for further investigating the functions of *NCED* genes in different species.

### 3.5. Genomic Landscape of Cis-Acting Elements in TaNCED Members

**Figure 4:**
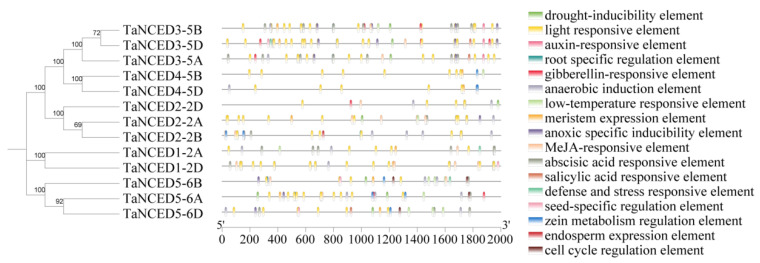
Analysis of *cis*-acting elements in *TaNCED* members. Boxes of different colors represent distinct *cis*-acting elements, with their names listed on the right.

To elucidate the potential regulatory landscape governing *TaNCED* expression, we comprehensively analyzed *cis*-acting elements in the promoter regions of *TaNCED* members using PlantCARE. This revealed an exceptionally rich repertoire of functional elements (Figure 4), strongly indicative of multifaceted roles for *TaNCED* genes beyond core ABA biosynthesis. We identified multiple regulatory elements associated with developmental regulation, such as light responsive element, root specific regulation element, meristem expression element, seed-specific regulation element, zein metabolism regulation element, endosperm expression element, and cell cycle regulation element. This pervasive presence of developmental regulators strongly implicates TaNCED proteins may participate in integrating ABA signaling with a broad spectrum of critical growth and developmental process throughout the wheat lifecycle, from germination to reproductive development. Furthermore, the promoter regions of *TaNCED* genes contain various phytohormone-responsive elements, including auxin-responsive element, gibberellin-responsive element, MeJA-responsive element, abscisic acid responsive element, and salicylic acid responsive element. The abundance of these elements, particularly ABA (potentially autoregulatory), MeJA, and SA, suggests intricate cross-talk between ABA biosynthesis/signaling and other major hormonal pathways. This positions *TaNCEDs* as potential nodes for integrating hormonal cues influencing stress responses, growth-defense trade-offs, and developmental transitions. Our analysis also predicted stress-related *cis*-acting elements, including drought-inducibility element, anaerobic induction element, low-temperature responsive element, anoxic specific inducibility element and defense and stress responsive element. This finding is highly significant, directly linking *TaNCED* expression to abiotic and biotic stress perception pathways. It strongly supports the hypothesis that ABA biosynthesis, mediated by specific TaNCED proteins, is a central hub in wheat’s adaptive responses to environmental challenges. Collectively, the phylogenetic grouping, structural diversity, and exceptionally complex promoter architecture provide compelling evidence for significant functional diversification within the wheat *TaNCED* members. These genes appear poised to integrate a wide array of developmental, hormonal, and environmental signals, thereby modulating ABA levels to fine-tune plant growth, development, and stress resilience.

### 3.6. TaNCED Members Exhibit Differential Spatiotemporal Expression During Vernalization

**Figure 5:**
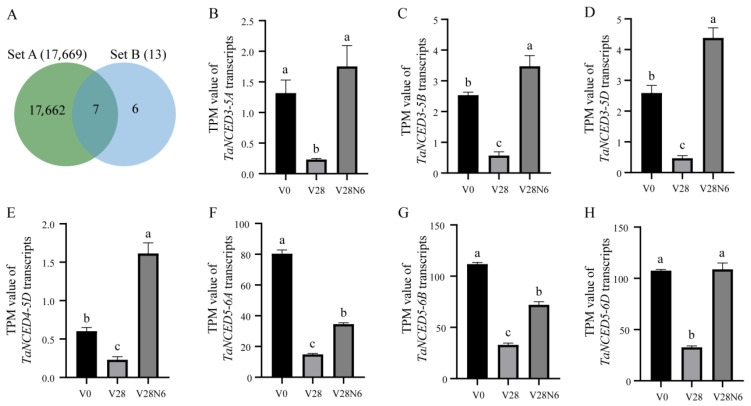
(**A**) Venn diagram showing seven *TaNCED* members associated with wheat vernalization response, with all the entire aerial parts of AK58 seedlings (including leaves, shoot, and shoot apical meristem) from different treatments but with synchronized developmental status at three-leaf stages [40]. Set A represents differentially expressed genes (DEGs) associated with wheat vernalization response. Set B represents *TaNCED* members. (**B**–**H**) The expression of *TaNCED3-5A* (**B**), *TaNCED3-5B* (**C**), *TaNCED3-5D* (**D**) *TaNCED4-5D* (**E**), *TaNCED5-6A* (**F**), *TaNCED5-6B* (**G**), and *TaNCED5-6D* (**H**). Data are mean ± *SD* (*n* = 3) are shown. Different lowercase letters above bars indicate significant differences among groups (*p* < 0.05), as determined using one-way ANOVA with Tukey’s multiple range test.

**Figure 6:**
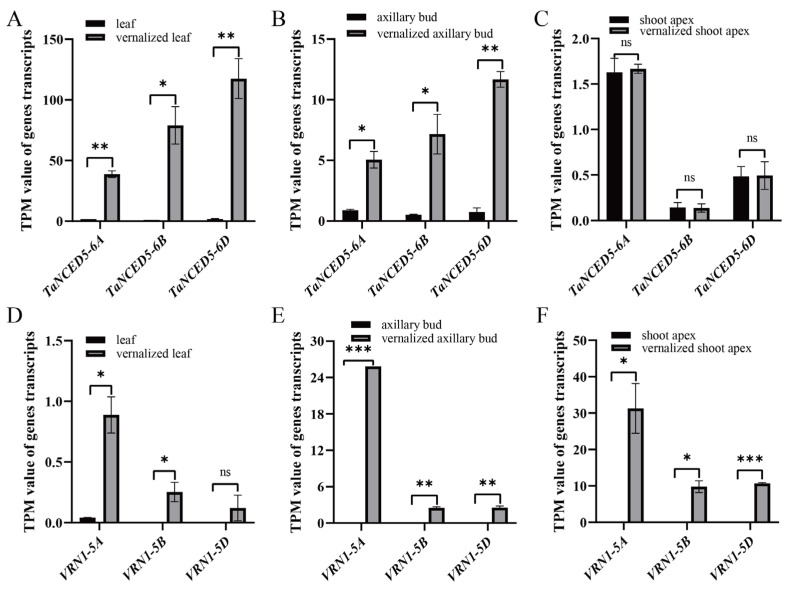
The expression of *TaNCED5-6A/6B/6D* and *VRN1-5A/5B/5D* in leaf, vernalized leaf, axillary bud, vernalized axillary bud, shoot apex, and vernalized shoot apex, respectively. (**A**) *TaNCED5-6A*, (**B**) *TaNCED5-6B*, (**C**) *TaNCED5-6D*, (**D**) *VRN1-5A*, (**E**) *VRN1-5B*, and (**F**) *VRN1-5D*. Data are mean ± *SD* (*n* = 2) are shown. ns, no significant difference; *, *p* < 0.05; **, *p* < 0.01; ***, *p* < 0.001, as determined using a two-tailed unpaired Student’s *t* test.

To dissect the roles of *TaNCED* gene family members in wheat vernalization response, we analyzed the expression of *TaNCED* gene family members using public wheat transcriptome datasets from vernalization experiments [40,41]. Our analysis uncovered striking distinct and tissue-specific expression patterns in response to low-temperature vernalization. The Venn diagram depicts 7 overlapping genes between the 17,669 differentially expressed genes (DEGs) during the vernalization process and 13 *TaNCED* gene family members (Figure 5A–H). Transcript quantification using TPM (Transcripts Per Million) values demonstrated distinct expression patterns among *TaNCED* paralogs across three key developmental stages (V0, V28, V28N6). Notably, *TaNCED5-6A* (Figure 5F), *TaNCED5-6B* (Figure 5G), and *TaNCED5-6D* (Figure 5H) exhibit significant differential expression. Next, we analyzed the tissue-specific expression of *TaNCED5-6A*, *TaNCED5-6B*, and *TaNCED5-6D* in axillary buds, vernalized axillary buds, leaves, vernalized leaves, shoot apexes, and vernalized shoot apexes. The results showed that the expression of the *TaNCED5-6A* (Figure 6A), *TaNCED5-6B* (Figure 6B), and *TaNCED5-6D* (Figure 6C) was significantly induced in vernalized leaves and axillary buds compared to non-vernalized controls. This specific upregulation strongly suggests that the *TaNCED5* homoeologs may play a role in mediating ABA biosynthesis during vernalization, particularly in leaves and bud tissues.

**Figure 7:**
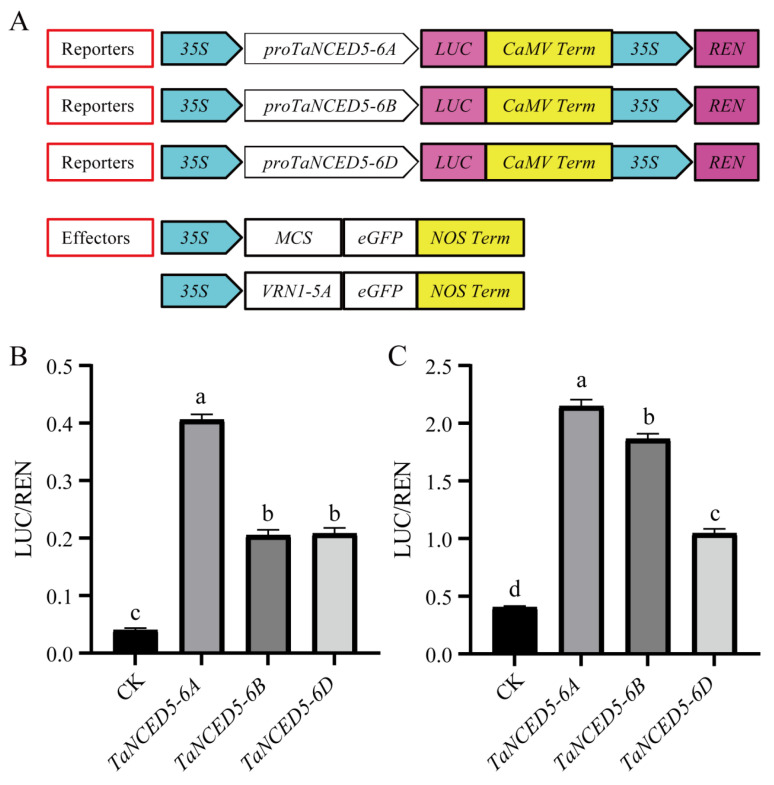
Schematic of dual-luciferase reporter assay system and transactivation analysis results. (**A**) Schematic diagrams of reporter and effector constructs. For reporters, the *35S* promoter drives the *proTaNCED5-6A*, *proTaNCED5-6B* and *proTaNCED5-6D* promoter sequence fused to the firefly luciferase (LUC) gene, with the *CaMV terminator* (*CaMV Term*) and a *35S-driven Renilla luciferase* (*REN*) gene as internal control. For effectors, the *35S* promoter drives either a multiple cloning site (MCS) with *eGFP* tag as CK and *NOS terminator* (*NOS Term*), or the *VRN1-5A* gene with *eGFP* tag and *NOS Term*. (**B**,**C**) Transactivation analysis results shown as the ratio of LUC to REN activity in Nicotiana benthamiana (**B**) and wheat (**C**) leaf protoplasts. Data are mean ± *SD* (*n* = 4) are shown. Different lowercase letters above bars indicate significant differences among groups (*p* < 0.05), as determined using one-way ANOVA with Tukey’s multiple range test.

Vernalization accelerates the transition of wheat from vegetative to reproductive growth by promoting the expression of the *VRN1* gene (Figure 6D–F). Wheat with incomplete vernalization exhibits characteristics such as an increase in the number of leaves and tillers, along with delayed heading/flowering date, thus raising the interesting question of whether *VRN1*, along with *TaNCED5*, may play a role in vernalization-mediated vegetative growth of wheat. Dual-luciferase reporter assays showed that VRN1 can activate the expression of *TaNCED5-6A*, *TaNCED5-6B*, and *TaNCED5-6D* (Figure 7A–C), suggesting that the inhibition of leaf and tiller production by low-temperature vernalization may be due to vernalization enhances the expression of *VRN1*, which in turn promotes the expression of *TaNCED5-6A*, *TaNCED5-6B*, and *TaNCED5-6D* in tiller buds and leaves, thereby facilitating the accumulation of ABA and inhibiting leaf production and bud dormancy.

Intriguingly, none of these members showed significant transcriptional changes in the shoot apical meristem during vernalization. This implies that the cold signal leading to vernalization in the meristem may not directly involve the transcriptional upregulation of *TaNCED* genes at this site. Overall, the differential regulation of *TaNCED5* homoeologs across tissues provides mechanistic insights into how wheat coordinates ABA-mediated stress responses with developmental phase transitions. The specific induction in leaves and buds positions these genes as potential integrators of environmental cold signals with growth regulation during vernalization.

## 4. Discussion

Abscisic acid (ABA) is a crucial phytohormone that plays a vital role in regulating plant responses to various environmental stresses, including drought, salinity, and cold [42,43,44], as well as in controlling plant development processes such as seed dormancy and germination [45,46]. The 9-*cis*-epoxycarotenoid dioxygenase (NCED) enzyme is a key rate-limiting enzyme in the ABA biosynthesis pathway, and its activity directly affects the level of ABA in plants. In this study, we conducted a comprehensive analysis of the *TaNCED* gene family in wheat, including identification, molecular features, phylogenetic relationships, conserved motifs and domains, gene structure, chromosomal distribution, collinearity, *cis*-acting elements, and expression patterns during vernalization. The results provide valuable insights into the evolutionary history, functional diversification, and regulatory mechanisms of the *TaNCED* gene family in wheat.

### 4.1. Structural Diversification and Functional Specialization of TaNCED Members

The comprehensive characterization of the *TaNCED* gene family reveals a sophisticated interplay between structural conservation and functional diversification. The 13 *TaNCED* members exhibit significant variation in amino acid composition, molecular weight, and isoelectric points, indicating potential differences in protein stability, solubility, and interactions with other molecules, which may be crucial for their distinct roles in ABA biosynthesis under different physiological conditions. For example, the relatively high instability index of some *TaNCED* proteins suggests that they may be subjected to rapid turnover, allowing for fine-tuning of ABA levels in response to changing environmental cues. The subcellular localization patterns of TaNCED members display a certain level of conservation. The exclusive chloroplast localization of all TaNCED members is consistent with the site of carotenoid biosynthesis, the precursor of ABA. The dual localization in chloroplasts and cytoplasm of TaNCED members imply a dynamic regulatory mechanism where ABA biosynthesis can be adjusted through subcellular shuttling, possibly in response to stress signals. Cytoplasmic localization of certain TaNCED proteins suggests their involvement in post-biosynthetic ABA signaling pathways or cytoplasmic regulatory interactions, highlighting the complexity of ABA metabolism and signaling in wheat.

The conservation of 10 motifs across most TaNCED members, with the notable exception of TaNCED1-2A lacking motif 6, suggests that these motifs are essential for the basic functions of NCED enzymes, such as carotenoid cleavage. The absence of motif 6 in TaNCED1-2A may lead to isoform-specific functional modifications, which warrants further investigation through site-directed mutagenesis to elucidate its specific role. The presence of the conserved RPE65 domain in all TaNCED members confirms their identity as functional NCED enzymes involved in ABA biosynthesis. The variation in gene structure, particularly the number of exons, indicates that gene duplication and rearrangement events have occurred during the evolution of the *TaNCED* gene family. This structural diversity may contribute to the functional divergence of *TaNCED* members, allowing them to respond to different developmental and environmental cues. For example, the three-exon structure of *TaNCED1-2A* may enable alternative splicing, generating different protein isoforms with distinct functions.

### 4.2. Regulatory Architecture Governing Spatiotemporal Expression

The analysis of *cis*-acting elements in the promoter regions of TaNCED genes reveals a complex regulatory network that integrates developmental, hormonal, and environmental signals. The presence of various developmental elements, such as light-responsive and meristem expression elements, suggests that *TaNCED* genes are involved in regulating ABA biosynthesis during different stages of plant growth and development. Hormone-responsive elements, including those for auxin, gibberellin, and ABA, indicate that *TaNCED* expression is tightly controlled by hormonal crosstalk, allowing plants to coordinate ABA levels with other phytohormones to balance growth and stress responses [47,48,49]. The identification of stress-related elements, such as drought-inducibility and low-temperature responsive elements, directly links *TaNCED* expression to abiotic stress perception pathways [50,51]. This finding supports the hypothesis that *TaNCED* genes play a central role in wheat’s adaptive responses to environmental challenges by modulating ABA biosynthesis. The intricate combination of these *cis*-acting elements in the promoters of different *TaNCED* members allows for precise spatiotemporal regulation of ABA levels, ensuring that plants can respond appropriately to changing environmental conditions.

### 4.3. Functional Implications for Vernalization Response

The differential expression patterns of *TaNCED* members during vernalization highlight their specific roles in this important developmental process. The significant upregulation of *TaNCED5-6A*, *TaNCED5-6B*, and *TaNCED5-6D* in vernalized leaves and vernalized axillary buds suggests that these genes are involved in mediating ABA biosynthesis in response to cold exposure. ABA is recognized to regulate cold acclimation and bud dormancy, with the induction of *TaNCED* genes potentially facilitating vernalization-mediated dormancy release and floral transition [52,53]. The lack of significant expression changes in the shoot apical meristem implies that ABA may be transported to this region from other tissues or that post-transcriptional regulation is more important in the meristem during vernalization. The tissue-specific expression patterns of *TaNCED* genes provide mechanistic insights into how wheat coordinates ABA-mediated stress responses with developmental phase transitions, ensuring that the plant can successfully undergo vernalization and flower under favorable conditions.

## 5. Conclusions

This study systematically identified and characterized 13 *TaNCED* gene family members in wheat. These genes are non-randomly distributed across chromosomes 2, 5, and 6. All TaNCED proteins harbor a typical conserved RPE65 domain, a catalytic hallmark essential for 9-*cis*-epoxycarotenoid cleavage in ABA biosynthesis. Notably, low-temperature vernalization treatment significantly induced the expression of *TaNCED5-6A*, *TaNCED5-6B*, and *TaNCED5-6D* in leaves and tiller buds. Collectively, these findings not only advance our understanding of *TaNCED* gene features but also provide valuable molecular information for enhancing climate resilience in wheat breeding programs.

## Figures and Tables

**Table 1:** Physicochemical parameters of *TaNCED* members.

Gene Name	AA (aa)	MW (Da)	pI	Instability Index	Aliphatic Index	GRAVY	Subcellular Localization
TaNCED1-2A	567	60,602	6.41	44.46	85.59	−0.039	Chloroplast
TaNCED1-2D	603	65,075	5.88	44.95	86.9	−0.049	Chloroplast
TaNCED2-2A	593	64,382	6.59	44.76	79.12	−0.307	Chloroplast and Cytoplasm
TaNCED2-2B	596	64,739	6.64	41.27	78.88	−0.288	Chloroplast and Cytoplasm
TaNCED2-2D	582	63,022	5.9	41.21	81.63	−0.244	Chloroplast and Cytoplasm
TaNCED3-5A	591	64,488	5.41	40.43	80.73	−0.193	Chloroplast and Cytoplasm
TaNCED3-5B	595	64,878	5.51	40.91	80.35	−0.195	Chloroplast, Cytoplasm and Mitochondrial
TaNCED3-5D	595	64,828	5.41	40.99	81.01	−0.176	Chloroplast and Cytoplasm
TaNCED4-5B	614	66,620	5.52	38.74	76.32	−0.2	Chloroplast and Cytoplasm
TaNCED4-5D	615	66,683	5.55	36.22	75.87	−0.2	Chloroplast and Cytoplasm
TaNCED5-6A	639	68,768	5.93	46.55	74.18	−0.207	Chloroplast and Cytoplasm
TaNCED5-6B	643	69,262	5.92	46.06	75.09	−0.194	Chloroplast and Cytoplasm
TaNCED5-6D	639	68,792	5.86	46.09	74.63	−0.201	Chloroplast and Cytoplasm

Abbreviations: AA (aa), Number of Amino Acid; MW (Da), Molecular Weight (Daltons); pI, Theoretical Isoelectronic Point; GRAVY, Grand Average of Hydropathicity.

## Data Availability

Data are contained within the article.

## Supplementary Materials

The following supporting information can be downloaded from https://www.mdpi.com/article/10.3390/biology14091293/s1, Figure S1: Conservative motifs logo of TaNCED members in wheat; Figure S2: Genomic collinearity analysis of *TaNCED* gene family members; Figure S3: Interspecific collinearity analysis of *NCED* gene family members; Figure S4: The collinearity of NCED gene family members among wheat and its ancestral species; Table S1: *NCED* members rename; Table S2: Primer sequences used in this study; Table S3: Tpm value of *TaNCED* members in different tissue, age and stress-disease; Table S4: KaKs analysis of *TaNCED* members; Table S5: Synteny analysis of *TaNCED* members.
