# Supplementary material for: Comprehensive Analysis of TaNCED Gene Family in Wheat Vernalization Process
Source: Biology (Basel). 2025 Sep 19;14(9):1293. doi: 10.3390/biology14091293 (PMC12467197; doi:10.3390/biology14091293)
Supplement: Supplementary file 1 [file biology-14-01293-s001.zip › Supplementary Figure S1 Conservative motifs logo of TaNCED members in wheat.pdf]

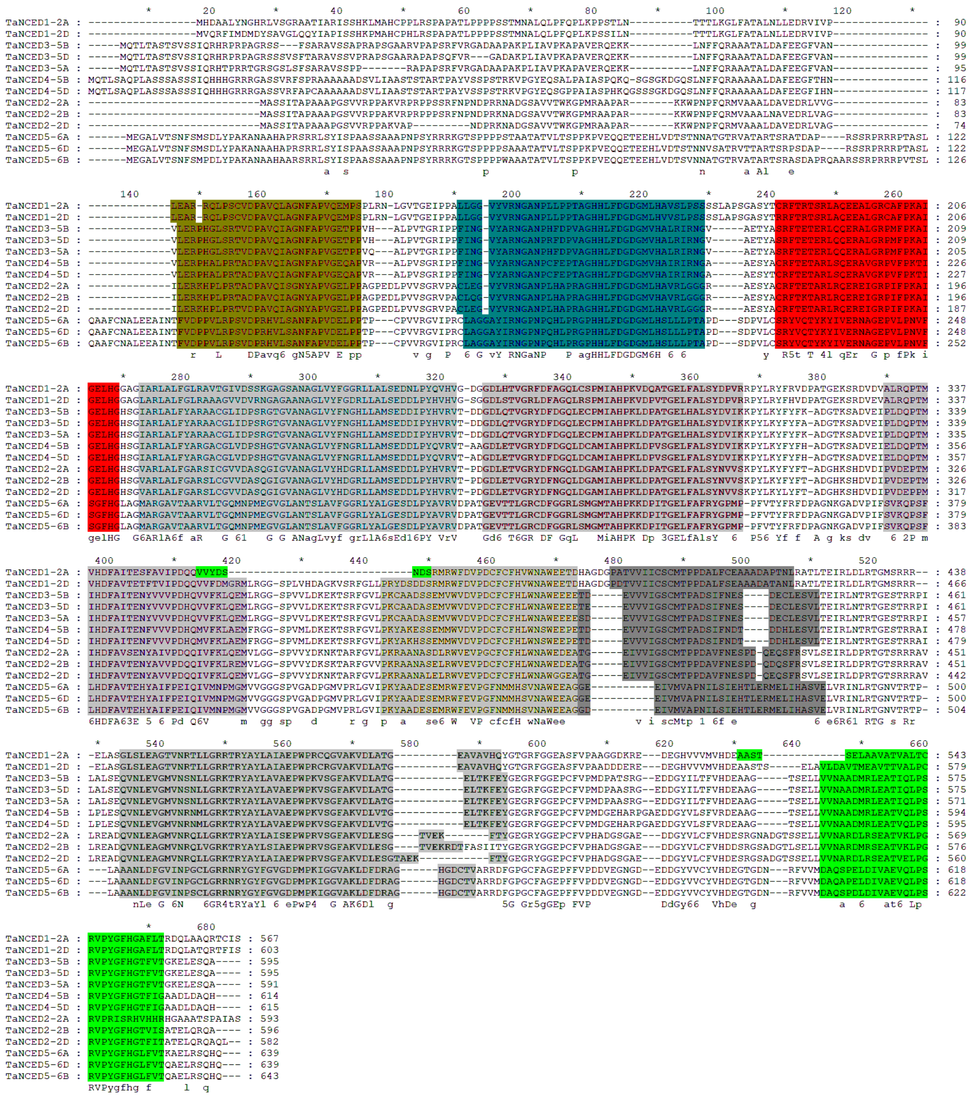

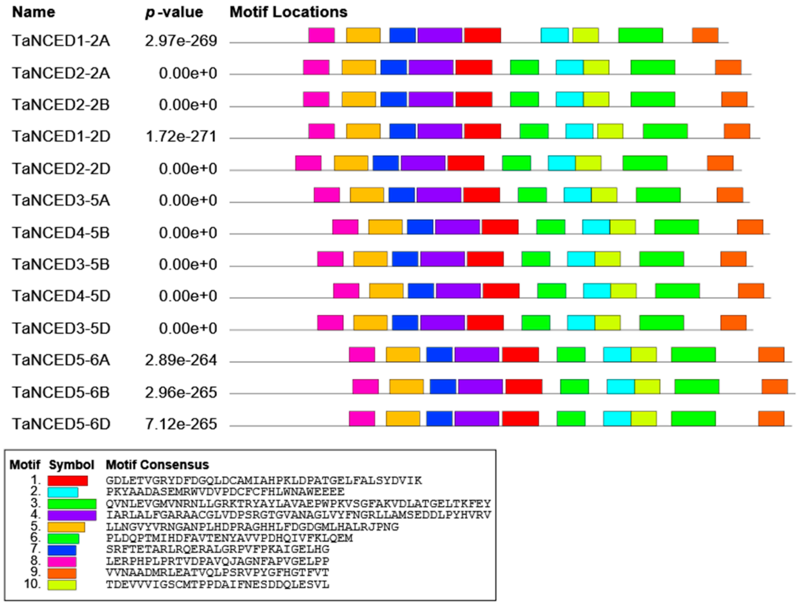

Supplementary Figure S1 The protein sequence alignment and conservative motifs logo of TaNCED members

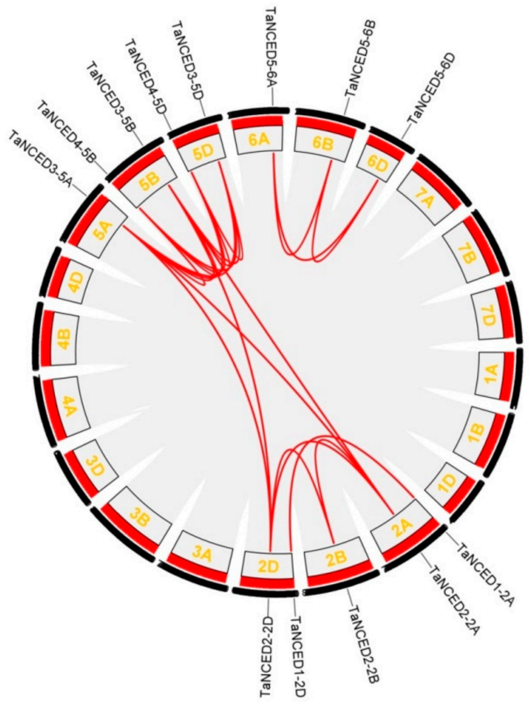

Supplementary Figure S2 Genomic collinearity analysis of *TaNCED* gene family members. Red lines indicate gene pairs with collinearity relationships,numbers within the boxes represent chromosomes, and red and light blue boxes denote gene density.

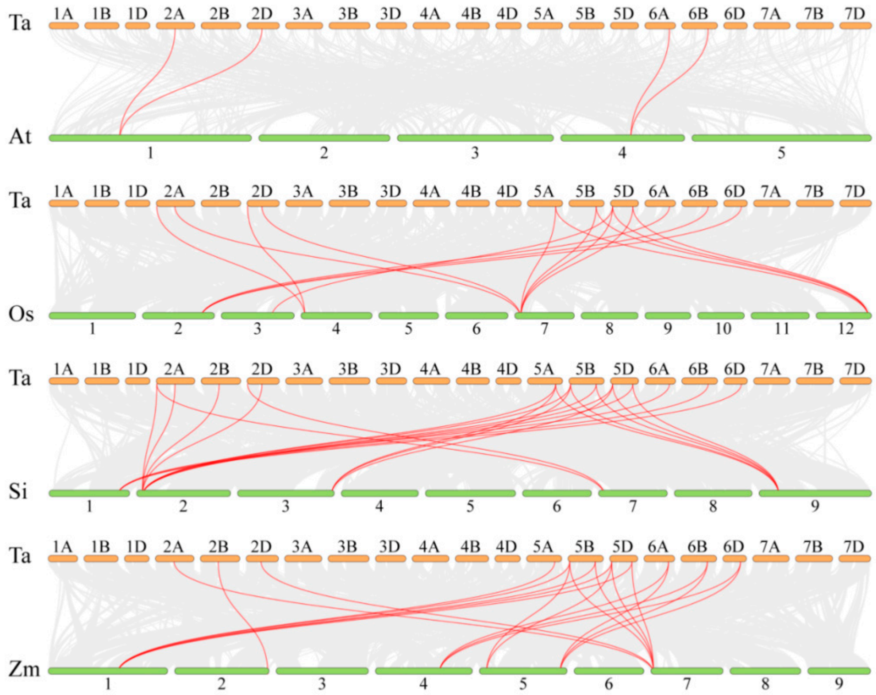

Supplementary Figure S3 Interspecific collinearity analysis of *NCED* gene family members. Red lines indicate collinear gene pairs between wheat (Ta) and other plant species, with chromosomal positions shown in circular layout. Species abbreviations: Ta, *Triticum aestivum* (wheat); At, *Arabidopsis thaliana*; Os, *Oryza sativa* (rice); Si, *Setaria italica* (foxtail millet); Zm, *Zea mays* (maize).

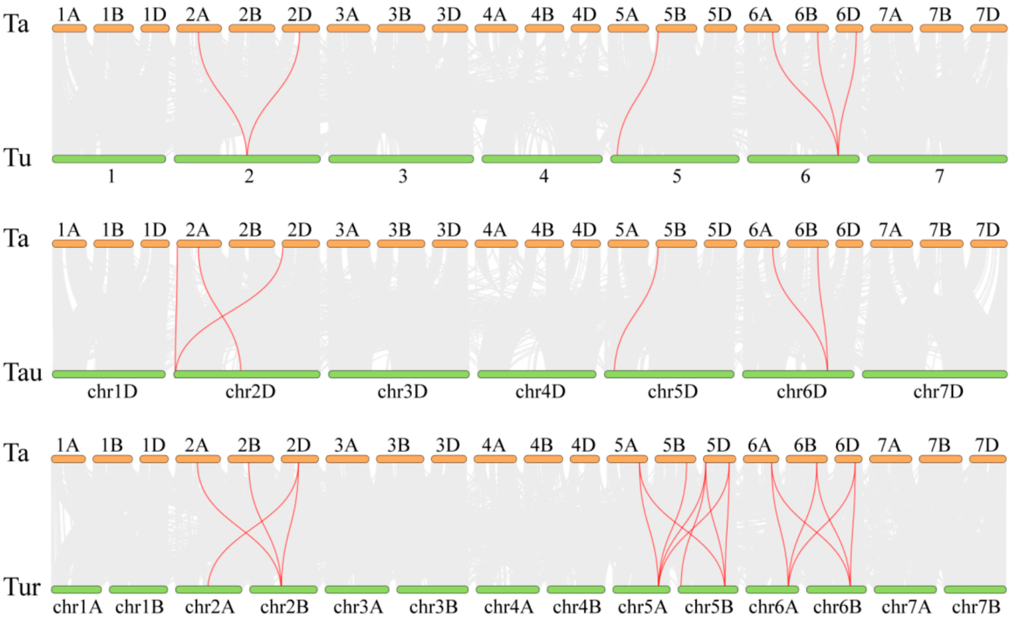

Supplementary Figure S4 The collinearity of *NCED* gene family members among wheat and its ancestral species.

Species abbreviations: Ta, *Triticum aestivum* (AABBDD); Tu, *Triticum urartu* (AA); Tau, *Aegilops tauschii* (DD); Tur, *Triticum turgidum* (AABB).
